# Primary intrathoracic malignant neurogenic tumor: report of three cases and comparison with benign neurogenic tumors resected at our institution

**DOI:** 10.1186/s40792-014-0013-1

**Published:** 2015-01-28

**Authors:** Takeshi Kawaguchi, Norikazu Kawai, Takashi Watanabe, Motoaki Yasukawa, Kohei Morita, Chiho Ohbayashi, Takashi Tojo

**Affiliations:** Department of Thoracic and Cardiovascular Surgery, Nara Medical University School of Medicine, 840 Shijo-cho, Kashihara, Nara 634-8522 Japan; Department of Diagnostic Pathology, Nara Medical University School of Medicine, 840 Shijo-cho, Kashihara, Nara 634-8522 Japan

**Keywords:** Mediastinal tumor, Malignant tumor, Neurogenic tumor, Surgery, Prognosis

## Abstract

We present three patients with intrathoracic malignant neurogenic tumor. Two lesions showed no sign of invasion into adjacent structures, while the third lesion extended to the intraspinal canal with vertebral involvement. Although all three lesions were completely excised, each patient relapsed within 1 year of the initial treatment. One patient with local recurrence underwent radiation therapy, but the recurrent tumor continued to progress. Chemotherapy was subsequently performed. Two patients with distant metastases also received chemotherapy. Because there is no effective chemotherapeutic regimen for intrathoracic malignant neurogenic tumor, all three patients received high-dose chemotherapy followed by hematopoietic stem cell transplantation. Although the relapsed lesions temporarily regressed after treatment, all three patients showed disease recrudescence and ultimately died of their disease. A comparison of the intrathoracic malignant neurogenic tumors and the benign neurogenic tumors resected at our institution revealed no meaningful differences distinguishing malignant from benign neurogenic tumors prior to surgery.

## Background

Neurogenic tumor is a common intrathoracic neoplasm, representing approximately 20% of all adult and 35% of all pediatric mediastinal neoplasms [1]. Among these cases, malignant neurogenic tumor (MNT) of the thorax is rare. Although its overall incidence remains unclear, it likely accounts for less than 1% to 2% of mediastinal neurogenic tumors [2]. In cases of MNT, radical surgical resection is necessary and is a positive prognostic factor; however, the overall survival is poor because of local and distant relapses. The utility of adjuvant chemotherapy or radiotherapy is unclear [1-6].

We report three cases of intrathoracic MNT treated with surgery. Additionally, we present a comparison of the clinical characteristics and outcomes of these patients and those of patients with benign neurogenic tumors (BNTs) resected at our institution.

## Case presentation

### Case 1

**Figure 1:**
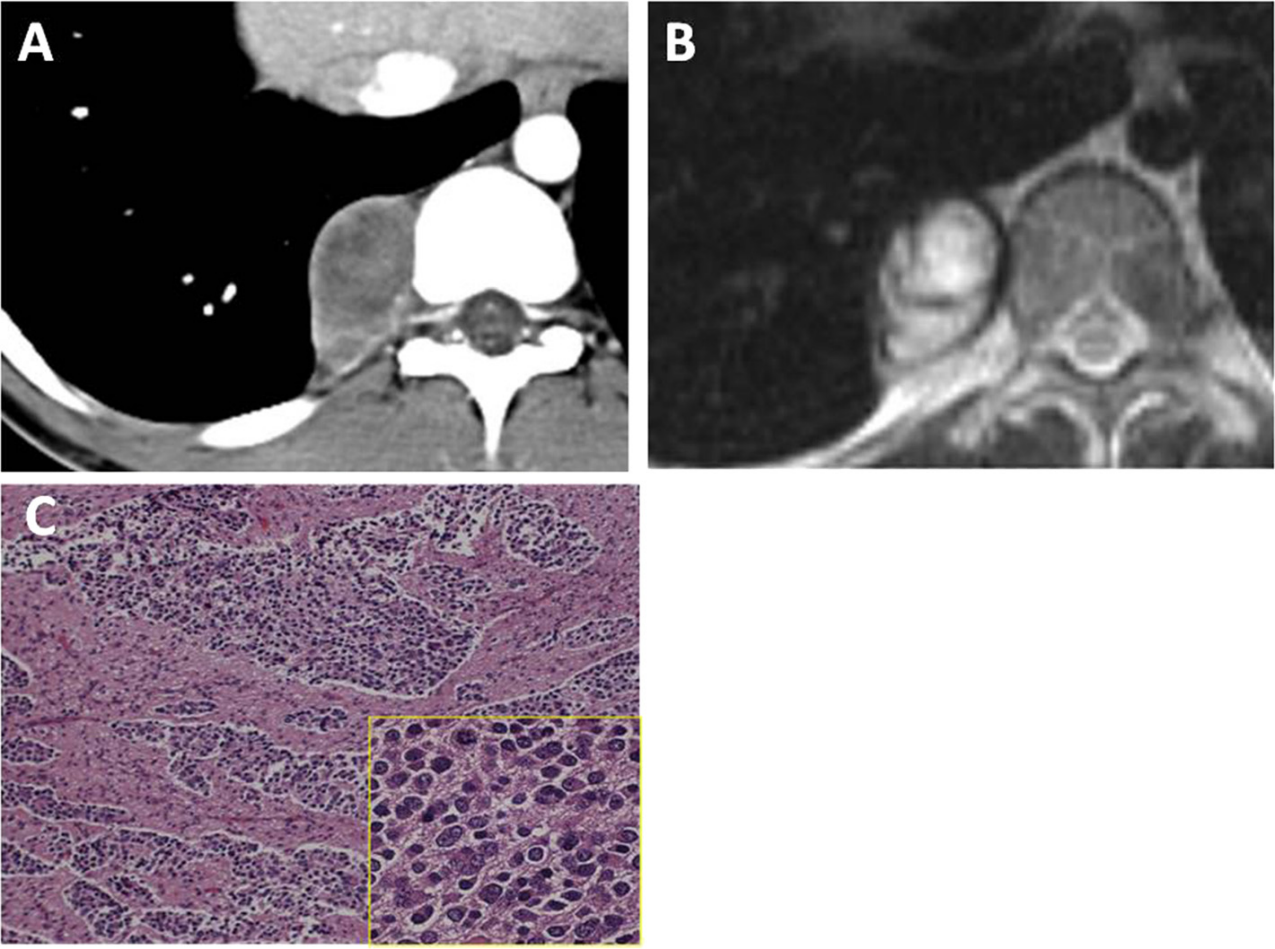
**Diagnostic exam results for case 1.** Chest enhanced CT **(A)** and T2-weighted MRI **(B)** showed a well-defined and ovoid mass located in the paravertebral sulcus without invasion of the vertebral body or intraspinal canal. The microscopic appearance of the area with a solid growth of primitive cells is shown **(C)**. The lesion was highly cellular (low-power view). The tumor nest was composed of primitive cells with round or oval hyperchromatic nuclei and scant cytoplasm (high-power view).

An abnormal shadow was detected on a chest radiograph in a 22-year-old male. Chest computed tomography (CT) and magnetic resonance imaging (MRI) revealed a posterior mediastinal tumor (Figure 1A,B). The patient was asymptomatic and had no signs of intraspinal canal extension on the imaging studies. He underwent surgical resection of the lesion. The operation was initially performed as video-assisted thoracic surgery (VATS), but the surgical approach was converted to a thoracotomy because the tumor was tightly attached to the chest wall. The tumor was excised completely (operative time: 2 h and 45 min; blood loss: 100 ml). Microscopically, the tumor consisted of two areas. One was a solid or isolated growth of oval primitive cells with Schwannian stroma, representative of a neuroblastoma (Figure 1C). The second was a diffuse growth of large polygonal cells with ganglion cell differentiation and prominent Schwannian stroma, which was regarded as a ganglioneuroma. Based on these characteristics, the tumor was diagnosed as a ganglioneuroblastoma.

The patient received postoperative radiotherapy as an adjuvant treatment, but it was discontinued halfway through when multiple bone metastases were identified. Subsequently, chemotherapy consisting of cisplatin (25 mg/m^2^ on days 1 to 5), cyclophosphamide (1,200 mg/m^2^ on days 1 and 2), vincristine (1.5 mg/m^2^ on day 1), and pirarubicin-doxorubicin (40 mg/m^2^ on day 3) was administered. However, progressive disease was demonstrated after 3 cycles of this regimen. Next, unrelated cord blood stem cell transplantation was carried out after a myeloablative conditioning regimen (etoposide: 500 mg/m^2^ on day −7; thiotepa: 180 mg/m^2^ on days −7, −6, and −5; total body irradiation: 2 Gy × 2 on days −3, −2, and −1). After this treatment, the bone metastases had regressed, and the patient was stable for approximately 1 year. Multiple bone metastases relapsed 18 months after the operation. High-dose chemotherapy comprising flutamide (30 mg/m^2^ on days −6, −5, and −4) and melpharan (100 mg/m^2^ on days −3 and −2) was performed followed by autologous peripheral blood stem cell transplantation (auto-PBSCT). Unfortunately, the treatment produced minimal response, and the patient died 24 months after surgery.

### Case 2

**Figure 2:**
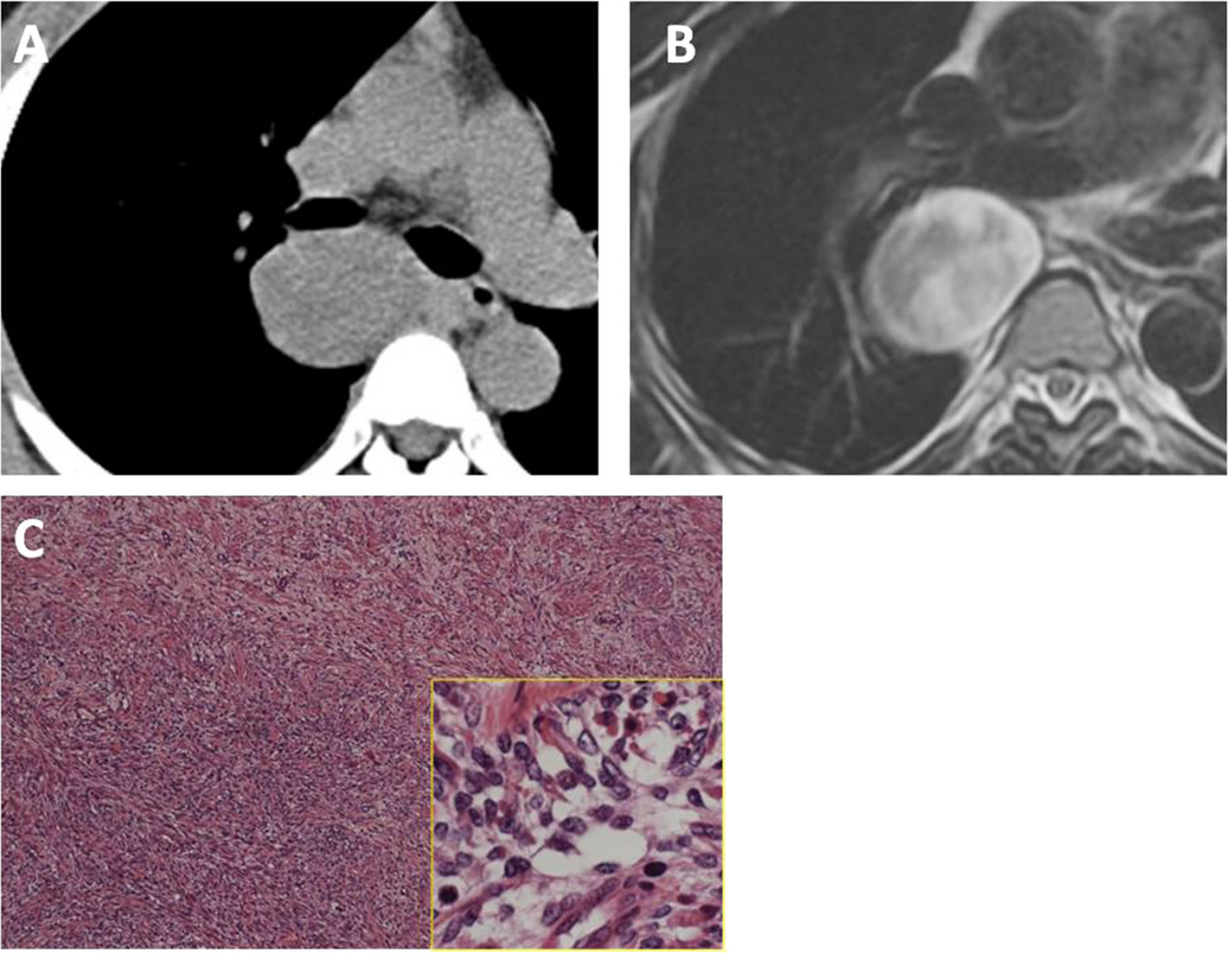
**Diagnostic exam results for case 2.** Chest unenhanced CT **(A)** and T2-weighted MRI **(B)** showed a well-defined and round mass located in the paravertebral sulcus without invasion of the vertebral body or intraspinal canal. The microscopic appearance of the tumor is shown **(C)**. It was a hypercellular, fascicular spindle cell neoplasm; the cells within the lesion had tapering nuclei and pale, indistinct cytoplasm (low-power view). Within these overtly malignant areas, numerous large, bright eosinophilic rhabdomyoblasts were seen (high-power view).

An abnormal shadow was detected on a chest radiograph in a 42-year-old female; a posterior mediastinal tumor was revealed on chest CT and MRI (Figure 2A,B). The patient was asymptomatic and did not suffer from neurofibromatosis type 1. She had no signs of intraspinal canal extension on the imaging studies. She underwent an operation via VATS. The tumor did not invade the surrounding organs and was completely excised (operative time: 3 h and 5 min; blood loss: 98 ml). Microscopically, the tumor consisted of spindle cells showing a fascicular growth pattern; they had wavy nuclei and eosinophilic cytoplasm. Within these overtly malignant areas, numerous rhabdomyoblastic cells and a neurofibroma region were seen (Figure 2C). On immunohistochemistry, spindle cells were positive for S-100 and negative for desmin, while rhabdoid cells were positive of desmin and myogenin and negative for S-100. Based on these histological and immunohistochemical features, a diagnosis of malignant peripheral nerve sheath tumor with heterologous rhabdomyoblastic differentiation was made.

The patient received postoperative radiotherapy as an adjuvant treatment (50 Gy). Multiple bone and lung metastases were revealed 14 months postoperatively. High-dose chemotherapy comprising carboplatin (400 mg/m^2^ on days −7, −6, −5, and −4), etoposide (15 mg/kg on days −5 and −4), and melpharan (90 mg/m^2^ on days −3 and −2) was performed followed by auto-PBSCT. After the treatment, the metastases regressed, and the patient was stable for 9 months. Lung metastases relapsed 25 months after the operation. Chemotherapy consisting of doxorubicin (20 mg/m^2^ on days 1 to 3) and ifosfamide (2 g/m^2^ on days 1 to 3) was administered. The regimen achieved a sustained partial response, and 5 cycles were performed, which reached the maximum dosage limit for doxorubicin. Progressive disease was noted 36 months postoperatively. Chemotherapy comprising dacarbazine (250 mg/m^2^ on day 1) and ifosfamide (2 g/m^2^ on days 1 to 3) was administered. However, the regimen did not produce a response, and the patient died 42 months postoperatively.

### Case 3

**Figure 3:**
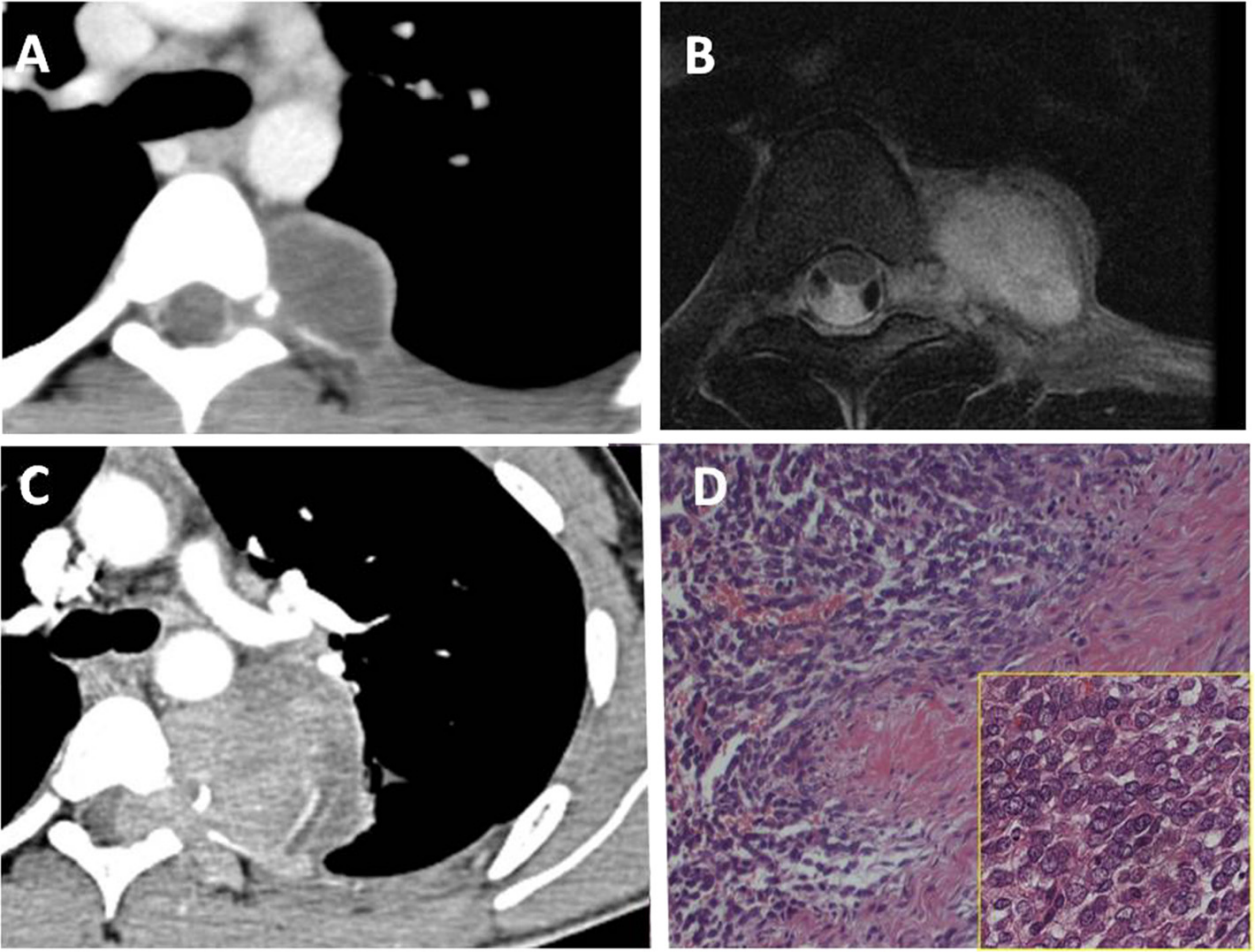
**Diagnostic exam results for case 3.** Chest enhanced CT **(A)** and T2-weighted MRI **(B)** showed a well-defined and ovoid mass located in the paravertebral sulcus. The tumor extended into the spinal canal. Six months after initial detection of the tumor, chest enhanced CT **(C)** showed a huge, heterogeneous mass extending into the intraspinal canal with involvement of adjacent vertebrae. The microscopic appearance of the tumor is shown **(D)**. The tumor was a highly cellular malignant spindle cell neoplasm that contained alternating cellular and more myxoid areas (low-power view). The tumor cells had eosinophilic cytoplasm, wavy hyperchromatic nuclei, and high mitotic activity (high-power view).

A posterior mediastinal tumor was detected on chest CT in a 17-year-old male without signs of neurofibromatosis type 1. Both chest CT and MRI revealed intraspinal canal extension (Figure 3A,B). Because the tumor was small and the patient was asymptomatic, the lesion was left untreated at the previous hospital. Six months after its initial detection, the tumor increased in size, and the patient was referred to our hospital. He was experiencing chest pain, and his chest CT showed a huge, heterogeneous mass extending to the intraspinal canal with involvement of adjacent vertebrae (Figure 3C). A CT-guided transthoracic biopsy of the tumor revealed malignant neurogenic tumor, and surgery was performed. First, a fourth to fifth hemilaminectomy was performed by the neurosurgical team. The tumor was released without spinal cord injury. Next, the intrathoracic component was removed via VATS performed by the thoracic team. The lesion was not disseminated, and pleural effusion was negative on intraoperative cytology. It was possible to completely remove the tumor with wedge resection of the adjacent lung (operative time: 8 h and 19 min; blood loss: 840 ml). Microscopically, the tumor was composed of short spindle cells showing a fascicular growth pattern; it had cellular and more myxoid areas. Polygonal cells were also seen. The tumor cells had eosinophilic cytoplasm, wavy hyperchromatic nuclei, and high mitotic activity (20 to 30 cells/10 HPF; Figure 3D). On immunohistochemistry, tumor cells were positive for S-100 and neurofilament and negative for actin, desmin, and keratin. Based on these morphologic features and the anatomic location, a diagnosis of high-grade malignant peripheral nerve sheath tumor was made.

The patient developed local recurrence 3 months postoperatively. Thoracic radiotherapy (60 Gy) was performed, but the recurrent tumor continued to progress; pleural dissemination developed. Subsequently, high-dose chemotherapy comprising carboplatin (400 mg/m^2^ on days −7 and −6), etoposide (15 mg/kg on days −7, −6, −5, and −4), and melpharan (90 mg/m^2^ on days −7 and −6) was performed, followed by auto-PBSCT. This treatment achieved a partial response, and for a time the patient remained stable with maintenance therapy of doxorubicin alone (50 mg/m^2^). Tumor regrowth was revealed 24 months postoperatively. At this time, high-dose chemotherapy with flutamide (30 mg/m^2^ on days −7, −6, −5, −4, −3, and −2), melpharan (70 mg/m^2^ on days −7 and −6), and ATG (2.5 mg/kg on days −5, −4, −3, and −2) was performed, followed by allogeneic peripheral blood stem cell transplantation. Although a partial response was temporarily achieved, the recurrent tumor grew again; the patient died 35 months after the operation.

### Comparison of patients with malignant and benign neurogenic tumors

Descriptions of the three patients with MNTs and the 21 patients with BNTs resected at our institution from 2000 to 2010 are provided in Table 1. Among clinical characteristics, no differences were seen between the two groups. In contrast, the prognosis for the two groups was completely different. All patients with BNT are alive without recurrence after resection, while three patients with MNT died of their disease.

**Table 1:** **Patient characteristics in the benign and malignant neurogenic tumor groups**

	**BNT (** ***n*** **= 21)**	**MNT (** ***n*** **= 3)**
Gender (male/female)	13/8	2/1
Age (years)	52 ± 18 (15 to 72)^a^	27 ± 14 (17 to 43)^a^
Symptom (yes/no)	2/19	1/2
Location	PM: 16	PM: 3
	Middle: 1	
	CW: 4	
Tumor size	33.9 ± 22.8 (11 to 90)^a^	43 ± 6.1 (30 to 50)^a^
Intraspinal extension (yes/no)	19/2	2/1
FDG-PET (positive/negative/not examined)	5/2/14	0/0/3
Surgical approach	VATS: 18	VATS: 1
	VATS + Post: 2	VATS → Open: 1
	Open: 1	VATS + Post: 1
Prognosis	Alive without Rec: 21	Died of tumor: 3

BNT, benign neurogenic tumor; MNT, malignant neurogenic tumor; PM, posterior mediastinal; Middle, middle mediastinal; CW, chest wall; VATS, video-assisted thoracic surgery; Post, posterior approach; Open, thoracotomy; Rec: recurrence. ^a^Mean ± standard deviation (range).

Three patients with MNT did not receive combined positron emission tomography/computed tomography using the tracer F-18-fluorodeoxyglucose (FDG-PET/CT) before operation. On the other hand, 7 patients out of 21 patients with BNT received FDG-PET/CT before operation. Among them, abnormal FDG uptake was revealed in five patients. Because the sample size was small and we did not have the data of MNT, we could not compare the FDG-PET/CT findings between MNT and BNT.

## Discussion

Neurogenic tumors are generally grouped into two categories: those of nerve sheath origin and those of sympathetic ganglia origin. Nerve sheath tumors are common in adults, while sympathetic ganglia tumors are common in children. Both types have malignant counterparts. Neurogenic tumors represent approximately 30% of all mediastinal neoplasms; most are BNTs, which have a good prognosis after resection [1]. Intrathoracic MNTs are rare, and few studies have described their clinical characteristics or outcomes.

We treated three patients with intrathoracic MNTs: two of nerve sheath origin and one of sympathetic ganglia origin. Although the three lesions were surgically excised, each patient relapsed within 1 year following the initial treatment. The first relapse was systemic in two patients and local in one patient. The patient with local recurrence underwent radiotherapy, which was unsuccessful in stopping the tumor growth. The other two patients received radiotherapy as an adjuvant treatment, but they had distant metastases and ultimately died of their disease. Generally, adjuvant radiotherapy is recommended after MNT resection to improve local control [4-6]. However, there is insufficient evidence regarding the efficacy of radiotherapy for MNT. While some papers have reported effective treatment with radiotherapy [7,8], it did not appear to improve the outcomes in our cases.

All three patients were treated with chemotherapy after recurrence was detected. There is no standard chemotherapeutic regimen for intrathoracic MNT, so the patients underwent chemotherapy based on regimens for neuroblastoma [9] or other malignant soft tissue tumors [10,11]. In all cases, their responses were insufficient. Subsequently, each patient underwent high-dose chemotherapy followed by hematopoietic stem cell transplantation (HSCT). George et al. previously reported long-term results from high-risk neuroblastoma cases treated with induction chemotherapy and local control measures followed by autologous PBSCT [12]; these authors concluded that tandem PBSCT could be safely performed in these patients and that it improved long-term survival. Unfortunately, although our patients’ relapsed lesions temporarily regressed after HSCT treatment, all three patients showed disease progression within 1 year of treatment and died of their disease. Previous reports have also demonstrated a poor prognosis with intrathoracic MNT [3,4,13,14]. Although complete resection is a necessary and potentially curative therapeutic modality for intrathoracic MNT, the prognosis following resection was unsatisfactory in our cases. In addition to local control, the establishment of a strategy to control systemic disease is required, as is the case with other high-risk soft tissue sarcomas.

The clinical characteristics of our three patients with intrathoracic MNTs were compared with those of patients with intrathoracic BNTs resected at our institution during the same time period (Table 1). Patients having MNT were younger compared with patients having BNT, although the BNT group also included a young adult patient. Other clinical features, including symptoms, tumor size, and invasiveness of the tumor, did not differ between the groups. Generally, greater tumor size, involvement of adjacent bony structures, and intraspinal extension are known to be signs of a MNT [1]. In the previously reported imaging analysis, size, surface characteristics, and internal heterogeneity of the lesion were reported to have predictive value of malignancy [15,16]. MRI has advantage in evaluating invasiveness to the neighboring organs and internal characteristics of the lesion. On the other hand, CT is superior in describing the tumor surface and vascularity. However, two of the three MNTs in this report were surgically excised without the combined resection of adjacent structures. Interestingly, the tumor with the most aggressive growth was the smallest in size among our three cases when detected. On the other hand, large-sized tumor or tumor with intraspinal extension was included in the BNT group of our study. In our experience with intrathoracic neurogenic tumors, we have not been able to differentiate MNTs from BNTs prior to resection.

Five of seven patients with BNT receiving FDG-PET/CT had abnormal FDG uptake in our study. Because the findings of FDG-PET/CT between MNT and BNT were not compared, we could not lead to the answer about the usefulness of the FDG-PET/CT. In the previous study, Cardona et al. reported the differences of the FDG uptake between BNT and MNT [17]. Although it may be useful to discriminate between MNT and BNT, further analysis is necessary.

## Conclusions

We have presented three cases with intrathoracic MNT. Although all three lesions were completely excised, each of the patients developed recurrence and ultimately died of the disease. The clinical differentiation of MNTs from BNTs was difficult before treatment.

## Consent

Before operation, we obtained general consent from every patient for using their clinical data for some clinical studies. However, the written informed consent for this case report was not obtained from the patients because this report is just retrospective case report without additional invasive examinations or treatments for the study.
